# A Case of Giant Aortoiliac Aneurysm Rupture when Open Repair Seems a One-Way Street

**DOI:** 10.1055/s-0040-1721747

**Published:** 2021-03-24

**Authors:** Georgios Galanopoulos, Vassilios Papavassiliou

**Affiliations:** 1 Department of Vascular Surgery, Metropolitan General Hospital, Athens, Greece; 2 Department of Vascular Surgery, Athens Medical Center, Athens, Greece; 3 Division of Vascular Surgery, Sismanoglio—Fleming Hospital of Athens, Athens, Greece

**Keywords:** aortoiliac, aneurysm, rupture, giant, open, repair

## Abstract

Giant aortoiliac aneurysm is a rare nosological entity. Owing to the increased diameter, the risk of rupture is extremely high and, similarly, the repair is extremely challenging. In this article, open surgical repair of a ruptured giant aortoiliac aneurysm in a 72-year-old male is described. A bifurcated Dacron graft was used with left internal iliac artery revascularization, while the contralateral internal iliac artery was ligated. The patient had an uneventful recovery.

## Introduction

The natural history of an aortoiliac aneurysm (AIA), as with every aneurysm, is progressive expansion and at the end, if not treated, rupture. Rupture is a catastrophic complication because it incurs an extremely high mortality rate. Most patients with ruptured AIA do not reach the hospital. Those who survive to be subjected to an intervention have an elevated mortality and morbidity rate. The annual risk of rupture increases with aneurysm expansion. It is calculated that the annual risk of rupture of an abdominal aortic aneurysm (AAA) with a diameter of >70 mm is 30 to 33%. 1 Ruptured AIA represents a special surgical challenge. Specific surgical skills are required. Giant AIAs are defined as those having a transverse diameter >13 cm. Herein, a case of a giant ruptured AIA treated successfully is described.

## Case Presentation

A 72-year-old man presented to the emergency department with pain in his right lower abdominal quadrant of 4 hours. His past medical history was significant for smoking (50 packs/year) and poorly controlled hypertension. He had no history of previous abdominal surgery. On admission, the patient had hypotension (84/46 mm Hg) and tachycardia (106 beats/min). Palpation of his abdomen revealed a large, tender pulsatile mass around the umbilicus.

**Fig. 1:**
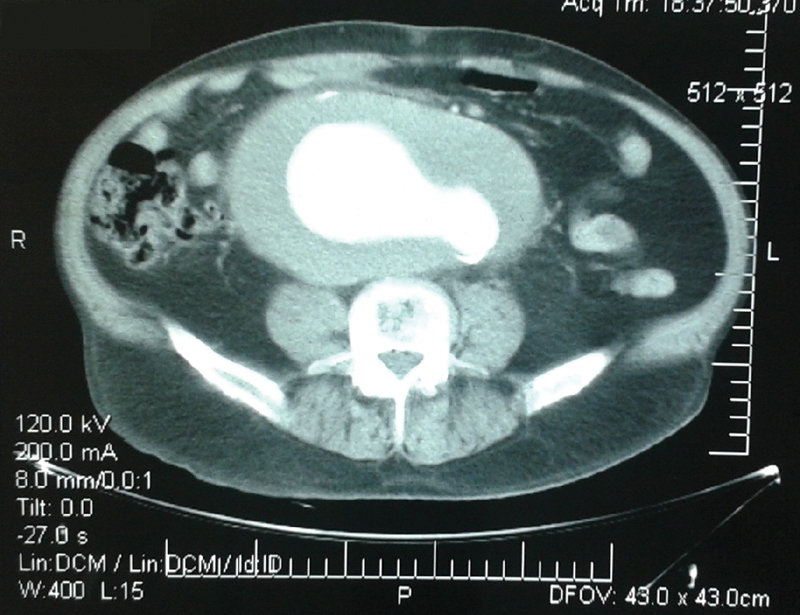
Contrast-enhanced computed tomography demonstrates a giant aortoiliac aneurysm at the level of the aortic bifurcation with extensive thrombus.

**Fig. 2:**
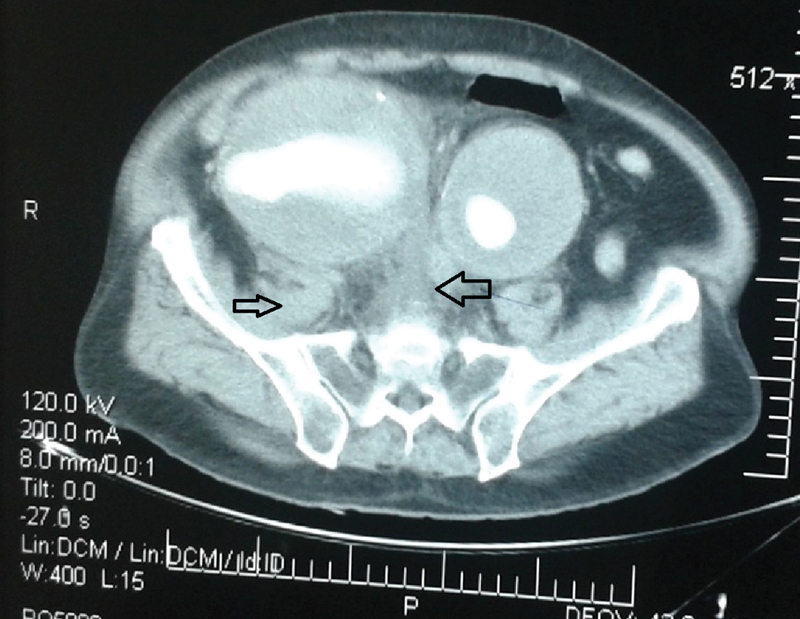
Appearance of giant iliac aneurysms with extensive thrombus and signs of rupture in the medial wall of the right iliac artery. Significant retroperitoneal pelvic hematoma is shown (arrows).

Emergency contrast-enhanced computed tomography revealed a giant infrarenal AAA extending to both common iliac arteries ( Fig. 1 ) with evident rupture at the medial wall of the right iliac aneurysm ( Fig. 2 ). This AIA measured 17 cm × 10 cm at the aortic bifurcation, 12 cm × 10 cm at the ruptured common right iliac artery aneurysm, and 9 cm × 8 cm at the left common iliac aneurysm. The aortic and both iliac aneurysms presented with extensive thrombus within the aneurysmal sacs. Both internal iliac arteries were patent.

The patient was immediately transferred to the operating theater.

An open transperitoneal approach was chosen. Through a midline incision, aortic cross-clamping was performed within a few minutes. Both external iliac arteries were dissected and controlled. Patent internal iliac arteries were controlled from within with inflatable balloons after opening the AIA. A **Y** -shaped Dacron graft 18 mm × 9 mm was used. Proximal anastomosis at the infrarenal aorta was performed just below the renal arteries. Distal anastomoses were performed in an end-to-end fashion with the external iliac arteries. The right internal iliac artery was oversewn from within at the ostium, while the left was revascularized via a jump graft from the iliac limb of the bifurcated graft. The jump graft was anastomosed in an end-to-end fashion to the ostium of the left internal iliac artery.

At the end of the procedure, the sigmoid colon had no signs of ischemia. During surgery, the patient was stable and received 8 units of packed red blood cells.

**Fig. 3:**
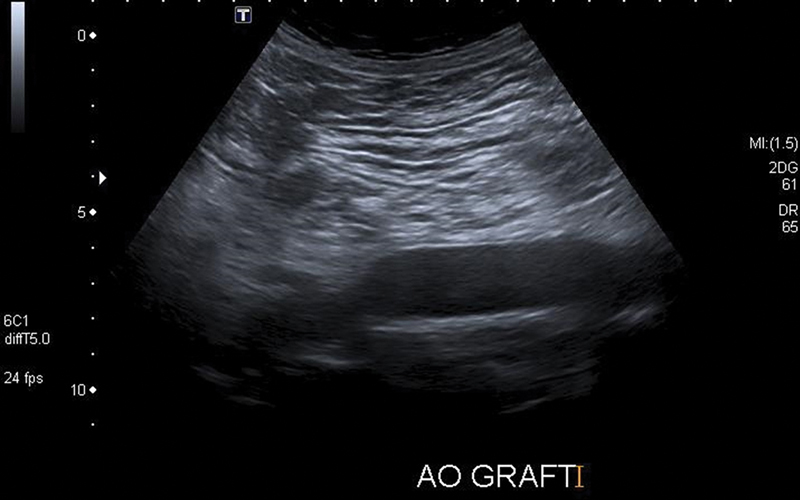
B-mode image of the proximal anastomosis.

**Fig. 4:**
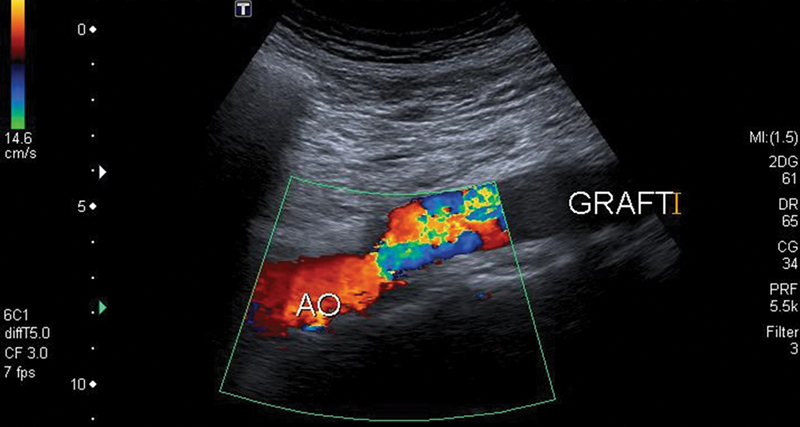
Color-flow Doppler image of the proximal anastomosis.

His postoperative course was uneventful and he was discharged from hospital on postoperative day 8. The patient continues to do well 3 years later. The last follow-up ultrasound imaging at 3 years from surgery revealed normal findings ( Figs. 3 and 4 ).

## Discussion

In the literature, giant AAAs occur almost exclusively in male patients. Clear predilection for male sex also characterizes aortic and iliac aneurysmal disease. The incidence of ruptured AIA seems to have declined in recent years, and this is partly due to the proliferation of screening in the population. The prevalence of giant AIAs is hard to estimate and is largely unknown. There are only a few reports in the literature, which means that giant AIAs are infrequently detected. The aforementioned screening protocols aim to timely detect AAAs in the population, recommending repair or surveillance according to their diameter, thus limiting the possibility for an aneurysm to excessively expand.

Although rupture rates are high for AAAs measuring >7 cm, strangely enough a small number of them, due to unidentified protective factors, do not rupture and continue to grow reaching extreme diameters.

The diameter and shape of the aneurysm are determining factors, closely related to the risk of rupture.

Sandhu and Pipinos 2 categorized iliac artery aneurysms based on their anatomical features. Combined aneurysms of the common iliac arteries and abdominal aorta are categorized as Type-E aneurysms and may be treated with bifurcated grafts.

The decision to take when facing with ruptured AIA concerns whether to treat it by open or endovascular means. Open repair is still the gold standard and always bears consideration in ruptured aneurysms. In experienced hands, complication rates are significantly reduced. Obviously, the presence of a giant AIA notably obfuscates the surgical field, making surgical maneuvers more demanding. Adjacent organs become adherent due to the extreme dimensions of the aneurysm, making dissection more difficult. On the other hand, endovascular repair has several limitations related to the anatomy of the diseased vessels, especially of the infrarenal aortic neck. Extreme angulation, large diameter, short length, and significant thrombus burden, all may accompany the extreme dimensions of the aneurysmal sac. 3 The anatomical suitability for endovascular treatment of ruptured AAA is reported at 46%. 4 Additionally, in the EUROSTAR (European collaborators on stent/graft techniques for aortic aneurysm repair) registry, larger (nonruptured) aneurysms were associated with increased incidence of endoleaks following endovascular repair, especially when there is concomitant aneurysmal pathology in abdominal aorta and iliac arteries. 5 In the IMPROVE trial (immediate management of patients with rupture: open versus endovascular repair), the 30-day mortality is similar among patients treated by open or endovascular means, with a shorter length of stay for those treated endovascularly. 6

The decision on how to treat a patient presenting to the emergency department with ruptured AIA is quite complex and multifactorial. This depends mainly on the experience of the operating doctor and his team. Special technical expertise is required. For both approaches if the experience is larger in one of the two treatment modalities, this is the case not to try the other one. If the availability of stent grafts, wires, catheters, balloons is limited, the endovascular way should not be attempted. Also, that endovascular treatment in emergency cases requires a readily available multidisciplinary staff with dedicated equipment. If there is no time to size the aneurysms and choose the best endograft, open repair is the only way.

In our particular case of giant ruptured AIA, the choice to immediately transfer the patient in the operating theater and obtain rapid aortic control represented an undoubtedly salvage procedure.

We are not aware of other reports of a giant aortic aneurysm measuring as much as 17 cm in transverse diameter combined with giant right and left common iliac aneurysms of as large as 12 and 9 cm, respectively.

The presence of bilateral common iliac artery aneurysms may be a limitation for endovascular treatment, due to the need for embolization of both internal iliac arteries to prevent retrograde flow and subsequently continuous bleeding. The risk of pelvic ischemia, with buttock claudication, bowel and urinary bladder ischemia, or erectile dysfunction, is high when both internal iliac arteries are sacrificed. On the other hand, the use of branched iliac devices combined with aortic bifurcated stent, grafts did not seem a safe option in our emergency situation. These procedures may be time consuming. Finally, it has been observed that concomitant aortic and common iliac aneurysms treated by endovascular means incur a high rate of endoleaks. 5 For this reason, prophylactic secondary interventions are frequently needed.

Open repair of AAAs may be related with several complications that become more prominent when dealing with giant AAAs. Significant displacement of adjacent organs enhances the risk of iatrogenic injuries especially when rupture of such aneurysms occurs. Venous injuries or small bowel serosal tears may also occur. To avoid such unpleasant surprises, cautious dissection after aortic cross clamping is mandatory, taking care to identify meticulously the surrounding anatomic structures. Additionally, pelvic revascularization in the presence of giant internal iliac aneurysms may be extremely challenging due to the limited surgical field in association with their location deep in the pelvis. Attempts to preserve at least one internal iliac artery should be made, as in our case.
